# Significant myocardial perfusion defect during stress visible in prone but not in supine imaging

**DOI:** 10.1007/s12350-022-03080-8

**Published:** 2022-08-17

**Authors:** Yvonne Andersson, Gabriela Fernandez, Peter Mars, Thomas Lindow

**Affiliations:** 1 Department of Clinical Physiology, Växjö Central Hospital, Region Kronoberg, Växjö, Sweden; 2 Clinical Physiology, Clinical Sciences, Lund University, Lund, Sweden

## Images that teach

In myocardial perfusion imaging, image artifacts due to soft-tissue attenuation can affect interpretation.^1^ Soft-tissue attenuation artifacts can be overcome using computed tomography attenuation correction or by acquiring images in both supine and prone position.^2–4^ Combined interpretation of supine and prone images improves diagnostic accuracy, in particular by improving specificity.^2,3,5^ Prone imaging is mainly used when acquiring stress images with the purpose to omit unnecessary rest images, by detecting attenuation artifacts.^6^ We present a case in which prone imaging instead helped to arrive at a diagnosis of significant myocardial ischemia.

**Figure 1:**
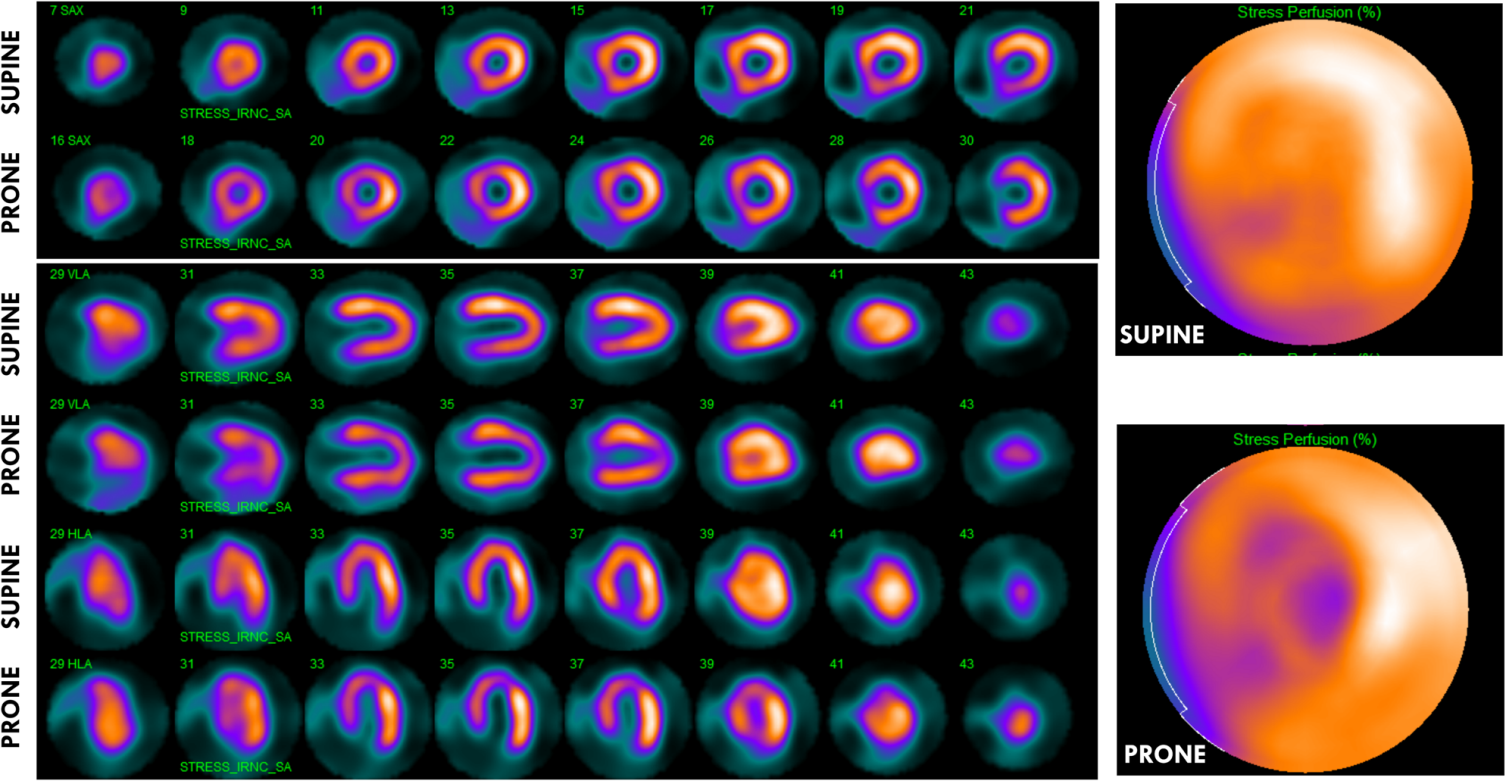
Myocardial perfusion images acquired by a cadmium zinc-telluride scanner after tetrofosmin technetium^99m^ injection during adenosine stress. Short- and long-axis images are presented to the left, and bullseye images to the right. In supine images a very mild relative uptake reduction was observed, while a significant defect in apical, septal, and anterior segments was found in prone images

**Figure 2:**
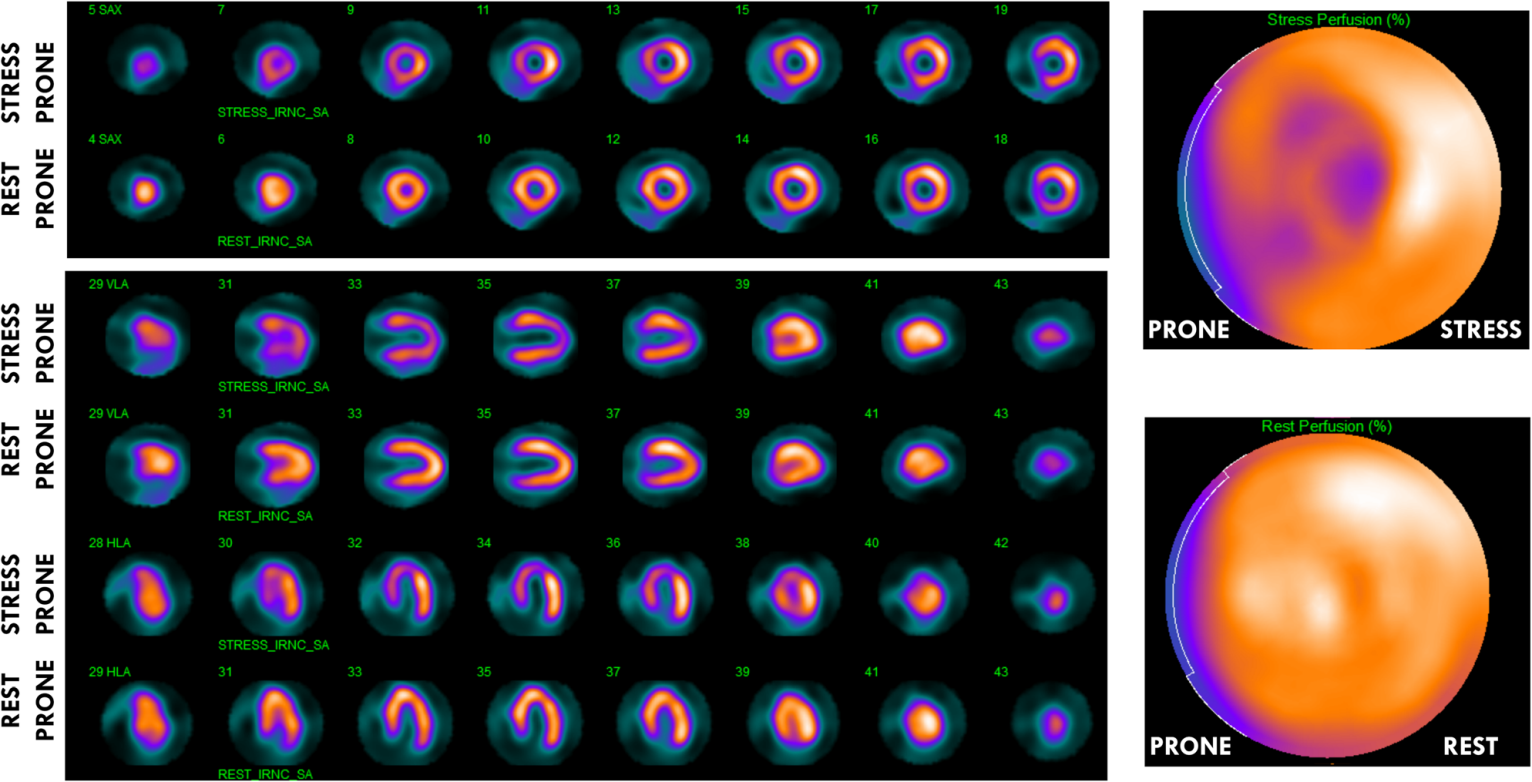
Prone images at stress and at rest showing normal rest perfusion and a perfusion defect during stress suggestive of stress-induced myocardial ischemia within the left anterior descending coronary artery territory

**Figure 3:**
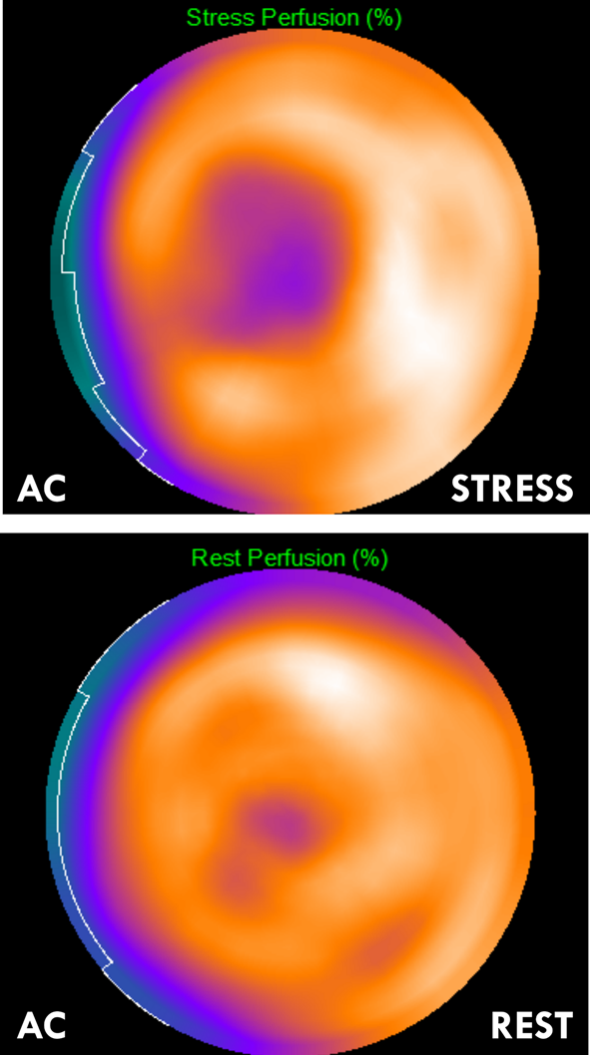
Fused computed tomography attenuation-corrected myocardial perfusion images (bullseye plots) showing a moderate stress-induced perfusion defect in the anteroseptal, apico-anterior, and apical segments of the left ventricle

**Figure 4:**
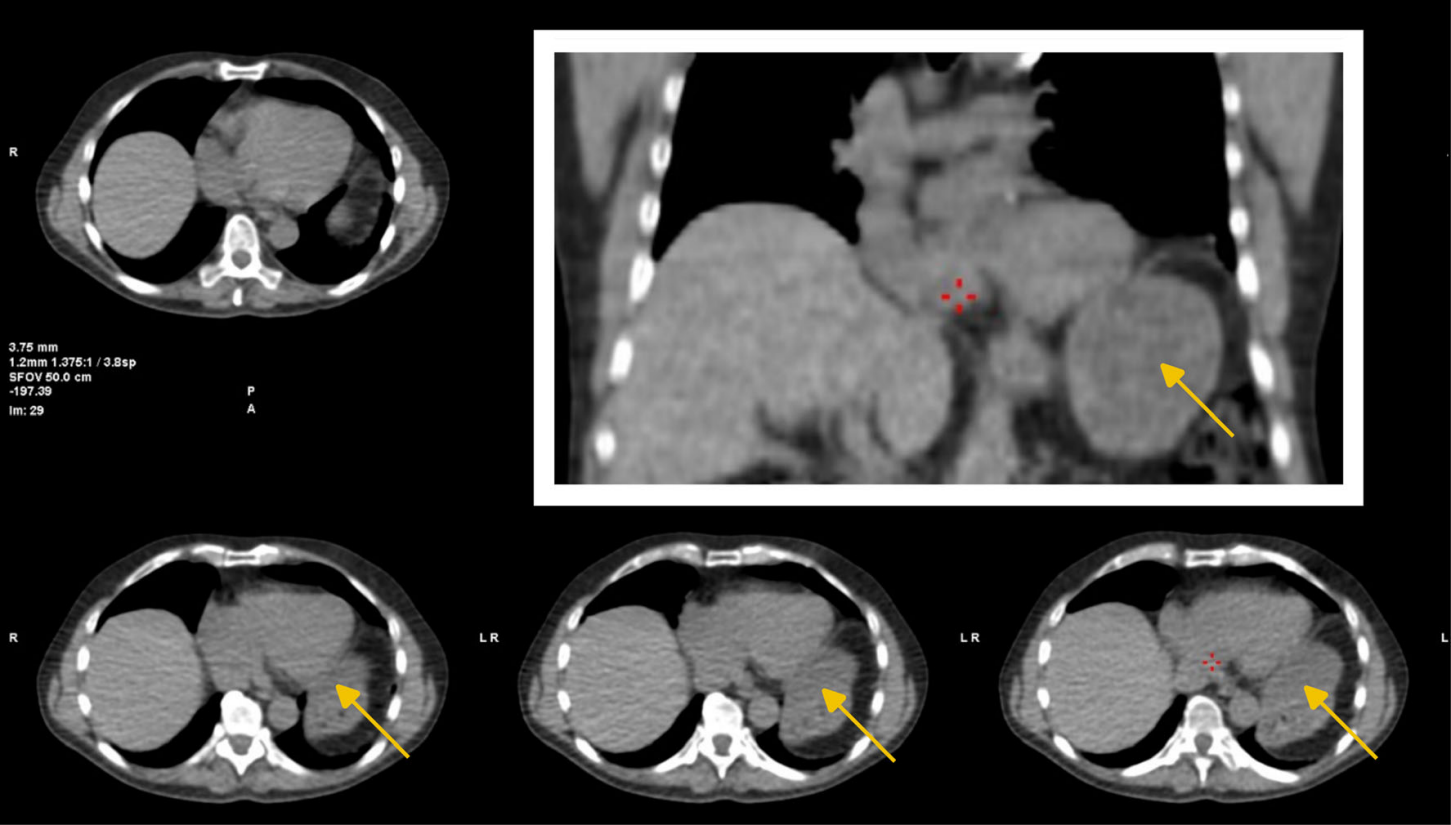
Low-dose computed tomography images (upper left and lower images: transversal slices; upper right image: coronal slice) used for attenuation correction showing an elevated diaphragm, which on the left side resulted in intestinal tissue (yellow arrows) being located in close proximity to the inferior and lateral left ventricular walls. This resulted in attenuation of normally perfused inferior and lateral walls and, in turn, regions with true defects to falsely appear relatively normal

A 76-year-old man with hypertension and hyperlipidemia reported exercise-induced chest pain and underwent a myocardial perfusion imaging test according to a 2-day protocol. Images acquired in supine position showed a near-normal isotope distribution, but prone images showed significant perfusion defects within left anterior descending coronary artery territory (Figure 1). Despite the near-normal supine images, a rest study was performed, including acquisition of images in prone position, which showed no perfusion defect (Figure 2), further raising the suspicion of significant exercise-induced ischemia. This was confirmed using fused computed tomography attenuation correction (Figure 3), which revealed an elevated diaphragm with intestinal tissue located close to the inferior/lateral walls of the left ventricle (Figure 4). Likely, this resulted in attenuation of the normally perfused inferior/lateral walls and a falsely normal relative perfusion of the anteroseptal and apical wall segments. Possibly, prone imaging shifted the relation between the intestinal tissue and the heart and consequently the attenuation conditions, thus revealing the true perfusion defect.

Presence of perfusion defects in prone images only may warrant additional imaging, e.g., by acquiring prone images at rest, or by the use of CT attenuation correction, for example when the perfusion defect in prone images corresponds to a typical coronary territory.

## References

[1] Hendel RC (2005). Attenuation correction: Eternal dilemma or real improvement?. Quart J Nucl Med Mol Imaging.

[2] Slomka PJ, Nishina H, Abidov A, Hayes SW, Friedman JD, Berman DS, Germano G (2007). Combined quantitative supine-prone myocardial perfusion SPECT improves detection of coronary artery disease and normalcy rates in women. J Nucl Cardiol.

[3] Taasan V, Wokhlu A, Taasan MV, Dusaj RS, Mehta A, Kraft S, Winchester D, Wymer D (2016). Comparative accuracy of supine-only and combined supine-prone myocardial perfusion imaging in men. J Nucl Cardiol.

[4] Mirshahvalad SA, Chavoshi M, Hekmat S (2022). Diagnostic performance of prone-only myocardial perfusion imaging versus coronary angiography in the detection of coronary artery disease: A systematic review and meta-analysis. J Nucl Cardiol.

[5] Nishiyama Y, Miyagawa M, Kawaguchi N, Nakamura M, Kido T, Kurata A, Kido T, Ogimoto A, Higaki J, Mochizuki T (2014). Combined supine and prone myocardial perfusion single-photon emission computed tomography with a cadmium zinc telluride camera for detection of coronary artery disease. Circ J.

[6] Trägårdh E, Valind S, Edenbrandt L (2013). Adding attenuation corrected images in myocardial perfusion imaging reduces the need for a rest study. BMC Med Imaging.

